# Characteristics of the initial patients hospitalized for COVID-19: a single-center report

**DOI:** 10.3906/sag-2004-98

**Published:** 2020-08-26

**Authors:** Alpay MEDETALİBEYOĞLU, Naci ŞENKAL, Gazi ÇAPAR, Murat KÖSE, Tufan TÜKEK

**Affiliations:** 1 Department of Internal Medicine, İstanbul Faculty of Medicine, İstanbul University, İstanbul Turkey

To the editors,

Novel coronavirus 19 infection (COVID-19) has become a global pandemic which originated in Wuhan, China, in December 2019 and caused worldwide mortality and morbidity [1]. Turkey had the first coronavirus patient tested positive on March 10th. Since then the number of tests and the number of patients with COVID-19 infection have increased and in less than 2 weeks the numbers climbed to thousands. In this analysis, we discussed the characteristics of patients admitted to our hospital for COVID-19.

We conducted a retrospective study among the patients who were hospitalized for the novel coronavirus in our internal medicine clinic between 15th and 28th March, 2020. Demographic characteristics and laboratory values of patients were recorded. Coronavirus testing was done with Bio-Speedy COVID-19 RT-qPCR kit. All the patients were also screened with chest X-ray and CT.

We evaluated 70 patients, 49 of whom were men (70.0%). Mean age was 55.8 (min 24, max 87). Thirtyseven patients (52.9%) tested positive for coronavirus infection with PCR. Patients who have negative PCR test results were also considered infected according to the radiological and clinical findings after other diagnoses are excluded with the viral respiratory panel. Seven patients (10.0%) had a recent travel history to disease-endemic countries, 29 (41.4%) had contact with suspected cases, and 10 (14.2%) were medical workers. Cough and fever were present in every patient except one (mean duration 3.7 and 3.5 days, respectively) and shortness of breath was present in 26 patients (37.1%) whereas myalgia was present in every patient (mean duration 0.7 days and 5.1 days, respectively). Twenty (28.6%) patients were smokers, 23 patients (32.9%) had hypertension (HT).

Among patients with HT, 6 (26.1%) were treated with angiotensin-converting enzyme (ACE) inhibitors, 8 (34.8%) with angiotensin receptor blockers (ARBs), 9 (39.1%) with calcium channel blockers, 5 (21.7%) with beta-blockers, 5 (21.7%) with thiazide diuretics and 2 (8.7%) with alpha-blockers. We continued hypertension medications during the hospital stay of the patients. There were 12 patients (17.1%) with diabetes, 6 patients (8.6%) with coronary heart disease, 2 patients (6.7%) with hematologic malignancy, and 3 patients (4.2%) with solid malignancy. Four patients had chronic obstructive pulmonary disease. Two patients (2%) were sent home because no hospital care was required. Eleven patients (15.7%), whose condition deteriorated, were transferred to the ICU. The remainders were followed in the clinic. There were statistically significant differences in SpO_2_, pulse rate, respiratory rate, hemoglobin, lymphocytes, monocytes, aspartate aminotransferase, lactate dehydrogenase, c-reactive protein, sodium, potassium, total protein, albumin, d-dimer, fibrinogen, activated partial thromboplastin time, troponin, and procalcitonin between these patients and patients followed in clinics (P < 0.05) (Table). There was no difference between parameters of patients with positive PCR and negative PCR results among the cohort.

**Table:** Comparison of patients in clinics and in intensive care unit.

	Patients followed in clinics (n = 57)	Patients followed in ICU (n = 11)	P-value
Age (years)	53.9 ± 14.4	70.1 ± 9.3	<0.001*
Sex	39 male (68.4%)	8 male (72.7%)	
SpO_2_ (%)	95.1 ± 2.7	88.3 ± 4.8	<0.001*
Pulse (per min)	95.7 ± 8.3	105.1 ± 7.7	0.002*
Respiratory Rate (per min)	19.0 ± 3.1	30.2 ± 3.2	<0.001*
pH	7.3 ± 0.0	7.4 ± 0.1	0.009*
pCO_2_ (mmHg)	42.8 ± 8.1	37.1 ± 10.9	0.022*
HCO_3_ (mmol/L)	24.8 ± 2.3	23.7 ± 3.2	0.299
Lactate (mEq/L)	1.5 ± 0,4	1.6 ± 0.8	0.556
Hemoglobin (g/dL)	13.4 ± 1.5	11.7 ± 1.9	0.015*
Platelets (per microliter)	186684.2 ± 56788.5	189545.4 ± 54869.5	0.877
WBC (per microliter)	5674.7 ± 1666.6	5578.1 ± 2580.0	0.907
Neutrophils (%)	4048.5 ± 1515.1	4662.7 ± 2623.4	0.901
Lymphocytes (%)	1047.1 ± 399.0	562.7 ± 284.7	<0.001*
Monocytes (%)	466.4 ± 181.5	308.1 ± 153.0	0.008*
BUN (mg/dL)	13.9 ± 6.1	21.3 ± 16.9	0.277
Creatinine (mg/dL)	0.9 ± 0.3	1.1 ± 0.7	0.609
Sodium (mEq/L)	137.2 ± 3.0	134.8 ± 4.4	0.08
Cl (mEq/L)	97.9 ± 3.0	95.9 ± 4.9	0.183
K (mEq/L)	4.3 ± 0.4	4.0 ± 0.4	0.044*
Glucose (mg/dL)	122.9 ± 30.1	128.4 ± 14.4	0.094
AST (U/L)	36.3 ± 21.1	64.8 ± 41.9	0.016*
ALT (U/L)	32.0 ± 25.9	37.5 ± 25.9	0.426
GGT (U/L)	33.3 ± 29.5	56.9 ± 47.7	0.102
ALP (U/L)	63.7 ± 20.2	81.0 ± 47.7	0.707
LDH (U/L)	299.4 ± 105.6	474.8 ± 244.1	0.008*
Total protein (g/dL)	7.1 ± 0.5	6.6 ± 0.4	0.003*
Albumin (g/dL)	3.9 ± 0.4	3.3 ± 0.3	<0.001*
CRP (mg/L)	50 ± 36.5	129.5 ± 69.2	<0.001*
Procalcitonin (ng/mL)	0.13 ± 0.2	0.3 ± 0.2	0.001*
Ferritin (ng/mL)	692.1 ± 891.8	818.3 ± 470.4	0.143
D-dimer (μg/mL)	816.6 ± 609.7	1950.0 ± 2033.9	0.002*
Troponin (pg/mL)	6.4 ± 6.5	27.0 ± 26.1	<0.001*
Pro-BNP (pg/mL)	133.7 ± 457.6	522.7 ± 584.9	<0.001*
Fibrinogen (mg/dL)	493.9 ± 89.1	582.9 ± 116.0	0.045*
APTT (seconds)	28.4 ± 2.7	34.9 ± 6.0	0.001*

WBC stands for white blood count, BUN for blood urea nitrogen, Cl for chloride, K for potassium, ALT for alanine transaminase, AST for aspartate transaminase, GGT for gamma-glutamyl transferase, ALP for alkaline phosphatase, LDH for lactate dehydrogenase, CRP for c-reactive protein, pro-BNP for pro-brain natriuretic peptide, APTT for activated partial thromboplastin time, and ICU for intensive care unit. Statistical analysis was conducted with SPSS v16. Data were tested for normality and parametric data was compared with Student’s t-test whereas nonparametric data was compared with the Mann–Whitney U test. Data were given with mean and standard deviation. A P-value less than 0.05 was identified as statistically significant.

**Figure 1:**
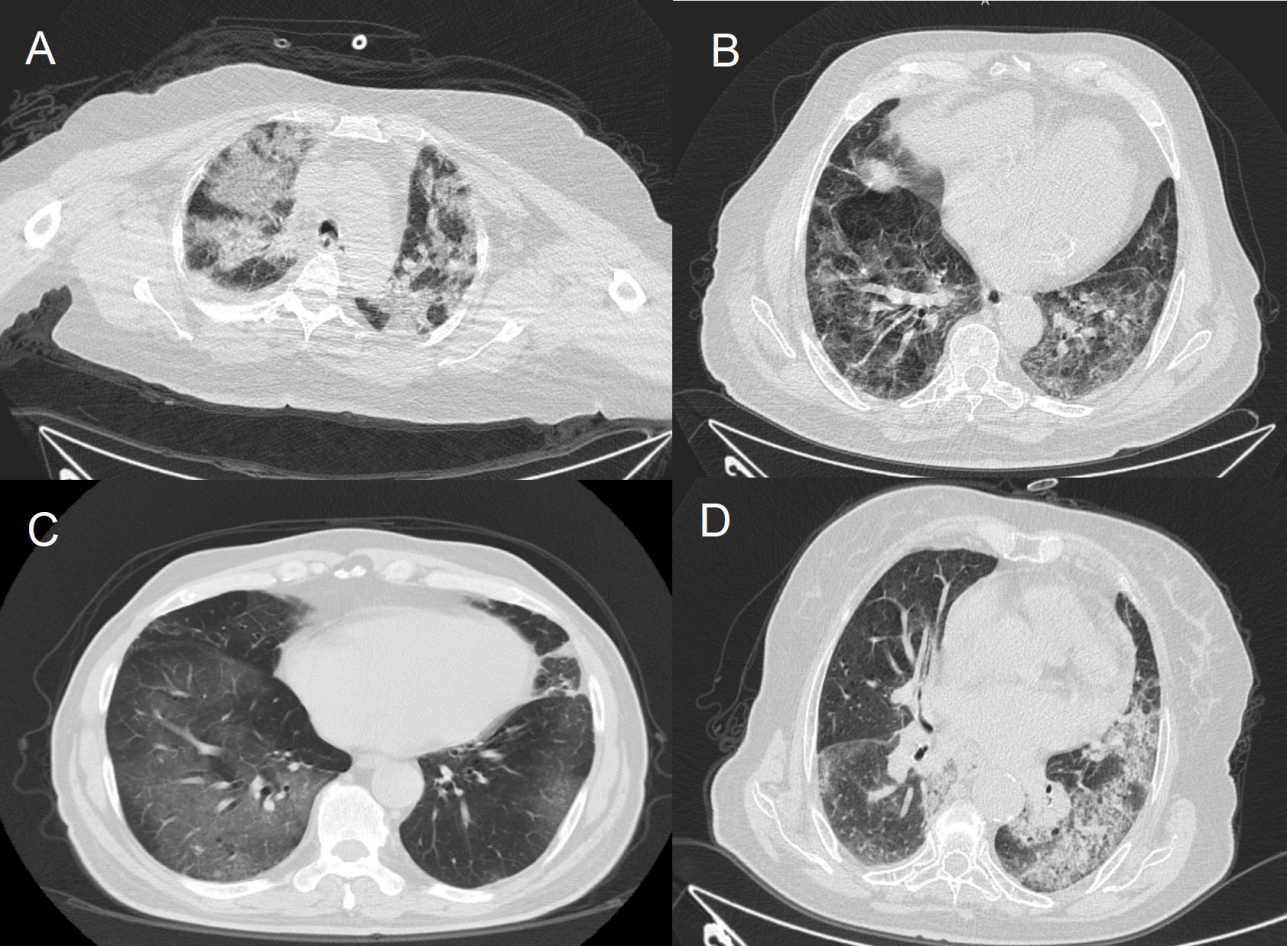
Computer tomography imaging of patients in intensive care unit.

**Figure 2:**
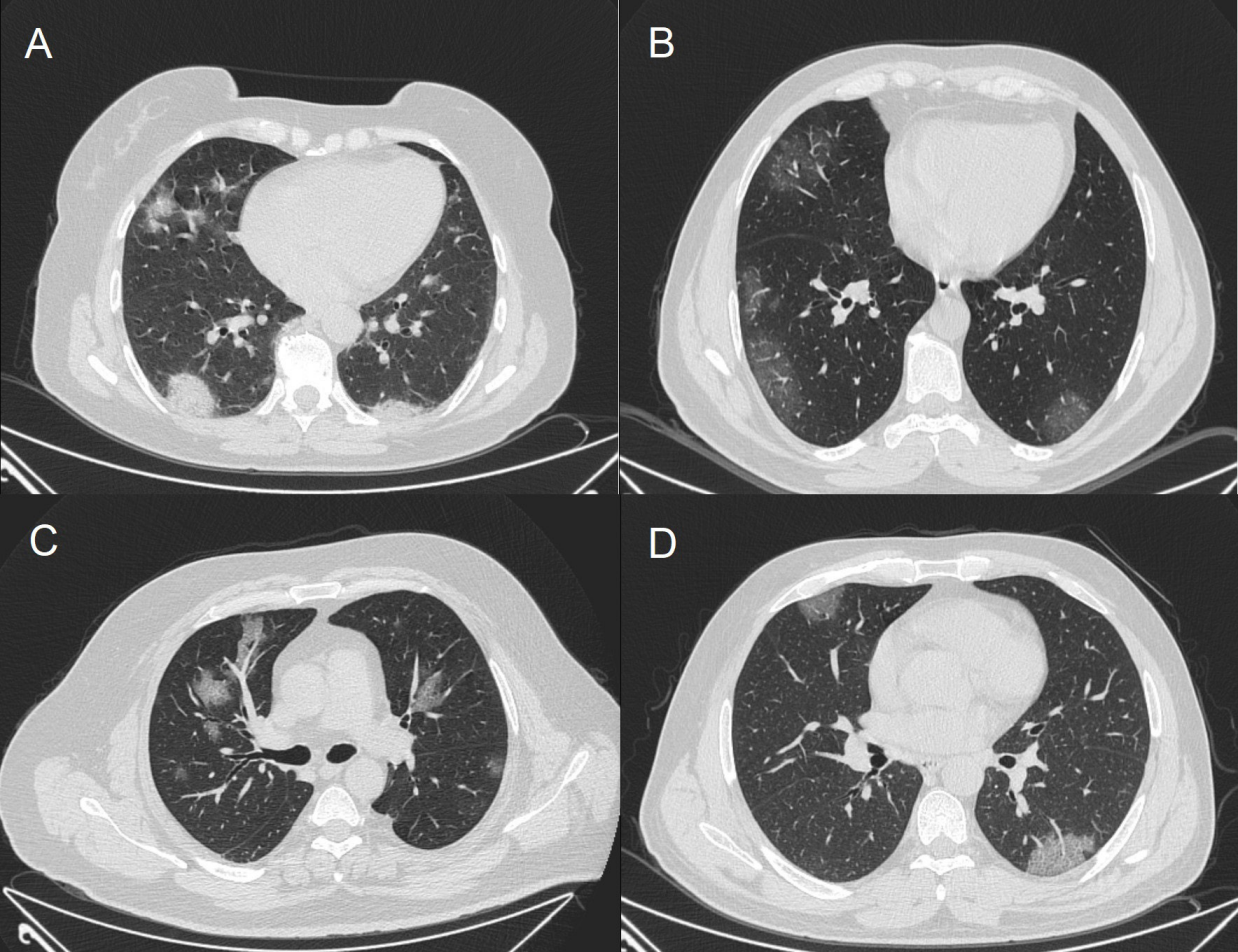
Computer tomography imaging of patients in clinics.

Chest CT scans showed infiltrations and ground-glass opacity. Patients admitted to ICU had more consolidated areas and infiltrations (Figure 1) than patients followed in clinics (Figure 2). All patients were started on hydroxychloroquine 2 × 200 mg, azithromycin 500 mg on day 1 followed by 250 mg for 4 days, ceftriaxone 1 × 2 g, and oseltamivir 2 × 75 mg. Other antiviral agents were added if patients’ condition deteriorated. Oxygen therapy was also administered to patients whose SpO_2_ was under 94%.

To the best of our knowledge, this is the first report of COVID-19 infection in Turkey. Numerous cohorts unite in one risk factor: age [2–4]. Li et al. reported the first 425 patients from Wuhan, China [5] and median age of their patients was 59. Our patients’ mean age was 55.8. Additionally, the mean age of patients treated in the ICU was 70, and it was statistically higher than that of patients treated in clinics. Zhou et al. [2] reported that age, d-dimer levels greater than 1 μg/mL, high-sensitivity cardiac troponin I, lactate dehydrogenase and lymphopenia were associated with severe disease. Our study results were compatible with those of this report.

Patients are mostly screened with RT-qPCR tests; however, there are publications of false-negative reports [6]. CT imaging of lungs also plays a crucial role in diagnosis [7]. Some reports say radiologic imaging has provided more insight for the detection of the disease and CT imaging is more reliable than PCR testing [7,8]. Nevertheless, clinical presentation and demographic features of the patients give clues about progression of the disease [9]. In our study, some patients whose radiology were compatible with COVID-19 but had negative PCR test results were assumed to be infected. 

In conclusion along with the strict preventive measures at the population level and the help of dedicated healthcare workers, we hope to defeat the novel coronavirus infection which is a completely unusual and severe threat to public health.
